# Life-Threatening Presentation of Pulmonary Hydatid Cyst with Hemoptysis and Hydatoptysis

**DOI:** 10.1590/0037-8682-0383-2024

**Published:** 2026-02-09

**Authors:** Omer Topaloglu, Elvan Senturk Topaloglu, Hasan Turut

**Affiliations:** 1 Recep Tayyip Erdoğan University Faculty of Medicine, Department of Thoracic Surgery, Rize, Turkey.; 2 Recep Tayyip Erdoğan University Faculty of Medicine, Department of Pulmonology, Rize, Turkey.

**FIGURE 1:**
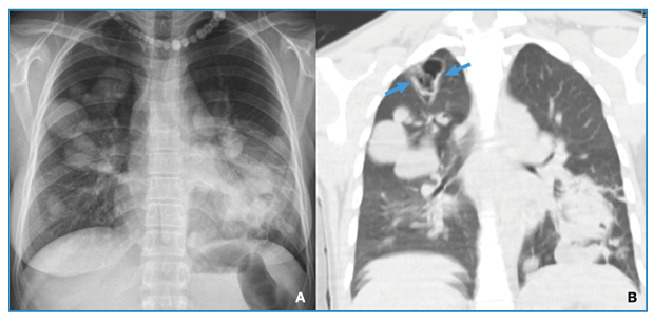
(A) Emergency posteroanterior chest radiograph showing diffuse cystic lesions in both lungs. (B) Thoracic CT scan depicting a ruptured cavitary lesion in the right apical upper lobe with evacuated intracystic fluid and visible fragments of the germinal membrane, which are most likely the source of hydatoptysis (blue arrows indicate the fragments).

**FIGURE 2:**
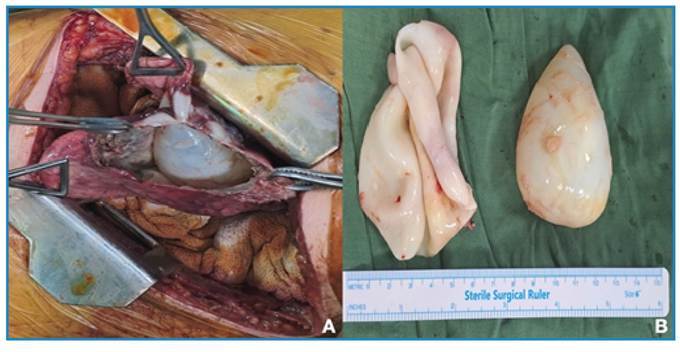
(A) Intraoperative image showing an unruptured hydatid cyst within the lung parenchyma. (B) Gross specimen demonstrating the germinal membrane and an enucleated cyst.

A 27-year-old woman presented to the emergency department with severe dyspnea, hemoptysis, expectoration of solid white fragments, and cough. Posteroanterior chest radiography revealed diffuse cystic lesions in both the lungs (Figure 1A). Computed tomography revealed a thin-walled cavitary lesion at the apex of the right upper lobe, heterogeneous cystic consolidation in the left lower lobe, and multiple parenchymal opacities, indicating pulmonary hydatid disease (Figure 1B). The indirect hemagglutination test returned positive results. Erosion of the cyst into the pulmonary vascular structures and its communication with the bronchi explained both hemoptysis and expectoration of salty/bitter water and whitish fragments (hydatoptysis). Subsequently, the patient underwent a staged thoracotomy. Multiple cysts were aspirated from both lungs and enucleated with their membranes; the cavities were then obliterated by capitonnage (Figure 2A). One cyst was removed via enucleation (Figure 2B). Albendazole therapy was initiated postoperatively.

Pulmonary hydatid disease remains an important health issue in endemic areas, particularly among young patients and may present as life-threatening respiratory symptoms^1^. Hemoptysis combined with hydatoptysis is a diagnostic sign of cystic rupture into the bronchi^1^. Early detection using chest imaging and prompt surgical treatment are essential to prevent fatal complications^2^. Additionally, early recognition of hemoptysis and hydatoptysis in young patients from endemic regions is crucial for implementing life-saving interventions.
